# Retraction: Melatonin protects spinal cord injury by up-regulating IGFBP3 through the improvement of microcirculation in a rat model

**DOI:** 10.1039/d1ra90007b

**Published:** 2021-01-20

**Authors:** Laura Fisher

**Affiliations:** Royal Society of Chemistry Thomas Graham House, Science Park, Milton Road Cambridge CB4 0WF UK advances-rsc@rsc.org

## Abstract

Retraction of ‘Melatonin protects spinal cord injury by up-regulating IGFBP3 through the improvement of microcirculation in a rat model’ by Kun Wang *et al.*, *RSC Adv.*, 2019, 32072–32080, DOI: 10.1039/C9RA04591K.

The Royal Society of Chemistry hereby wholly retracts this *RSC Advances* article due to concerns with the reliability of the data. The images in the article were screened by an image integrity expert. After processing the images, the expert was able to verify instances of duplicating images affecting Fig. 3A:

The EB/control and EB/SCI + melatonin panels are from the same image but shown at different magnifications.

The FITC-LEA/SCI and FITC-LEA/SCI + melatonin + IGFBP3 panels both contain part of the same image.

The morphology of the images in the EB/SCI and FITC-LEA/SCI + melatonin panels are very similar indicating that they could be based on the same duplicate image.

The authors were asked to provide the raw data for this article, but did not respond. Given the significance of the concerns about the validity of the data, and the lack of raw data, the findings presented in this paper are not reliable.

The authors have been informed but have not responded to any correspondence regarding the retraction.

Signed: Laura Fisher, Executive Editor, *RSC Advances*

Date: 7^th^ January 2021

## Supplementary Material

